# Genetic Evidence on the Role of Blood Phytosterols in Frailty: A Mendelian Randomization Study

**DOI:** 10.1002/fsn3.70616

**Published:** 2025-07-14

**Authors:** Guangyu Gao, Tianci Yao, Chengyun Liu, Haohui Fan, Xinyue Zhang, Hao Zhang, Xiaofang Zhao, Bei Song, Kun Wang, Ting Liu, Xueke Guang, Quan Zhou, Weilin Lu

**Affiliations:** ^1^ Department of Geriatrics, Union Hospital Tongji Medical College, Huazhong University of Science and Technology Wuhan China; ^2^ Department of Endocrinology Yueyang Central Hospital Yueyang China

**Keywords:** frailty, Mendelian randomization, phytosterol

## Abstract

Phytosterols have been recommended as a lifestyle intervention for early lipid management—which has a significant impact on frailty. However, their effect on frailty remains unclear. Studies have shown that genetic proxied total blood phytosterol affects the development of cardiovascular disease through non‐HDL‐c and apolipoprotein B mediation, which makes phytosterol an underlying risk factor for frailty. The aim of this Mendelian randomization (MR) study was to investigate the genetic associations between phytosterols and frailty. We used univariate Mendelian randomization (UVMR) to assess the causal effects of blood phytosterols on the Frailty Index (FI) and Fried Frailty Score (FFS). We also employed multivariate Mendelian randomization (MVMR) and Two‐step MR (TSMR) to evaluate the mediating role of blood lipids in the relationship between blood phytosterols and FI. We used the product of coefficients method to calculate the mediating effect. The inverse‐variance weighted method was used as the primary analysis. Genetically proxied higher levels of blood total sitosterol were significantly associated with a higher risk of Frailty Index (OR = 1.035, 95% CI = 1.009–1.061, *p* = 0.008). MVMR analysis revealed that the causal association between blood total sitosterol and Frailty Index was attenuated after adjusting for non‐HDL‐C and APOB. Non‐HDL‐C and APOB mediated 54.3% (21.1%, 87.5%) and 49.9% (17.7%, 82.1%) of the effect of blood phytosterols on the Frailty Index, respectively. No significant association between blood phytosterols and Fried Frailty Score was detected. This study suggests a modest association between blood phytosterols and an increased risk of FI. Furthermore, non‐HDL‐C and APOB may mediate a significant proportion of the association between blood sitosterol and FI. The differential effects of blood phytosterols on FI and FFS outcomes may indicate a mediating role of specific health deficits, particularly cardiometabolic factors.

## Graphical abstract

This Mendelian randomization study found genetically elevated blood sitosterol levels modestly increased Frailty Index risk (OR = 1.035, *p* = 0.008), with non‐HDL‐C and ApoB mediating ~50% of this effect. No association was observed with Fried Frailty Score, suggesting specificity to cardiometabolic deficits captured by the Frailty Index. Results imply phytosterols may influence frailty through lipid pathways, warranting caution in their therapeutic use for aging populations.

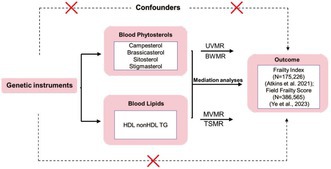

## 1 Introduction

Frailty is characterized by a decline in the physiological function of multiple systems (Clegg et al. 2013; Cheung et al. 2018; Fried et al. 2001), leading to increased vulnerability to stressors (Clegg et al. 2013). It is recognized as an emerging global health burden with significant implications for clinical practice and public health (Hoogendijk et al. 2019). Frailty can lead to adverse outcomes such as disability, falls, fractures, deterioration of mobility, loneliness, decreased quality of life, depression, cognitive decline, dementia, hospitalization, and nursing home admission (Hoogendijk et al. 2019). The prevalence of frailty is approximately 13% in the general population (Almohaisen et al. 2022; Veronese et al. 2021), 51.5% in nursing homes, and ranges from 21% to 43% in hospitalized patients based on cohort studies (Flaatten et al. 2017; Aliberti et al. 2021; Jung et al. 2021). Given the lack of effective pharmacological treatments for frailty (Dent et al. 2019), early identification and modification of risk factors in at‐risk populations are crucial to reduce the societal burden of this condition.

Frailty is commonly assessed using the Frailty Index (FI), a measure based on the accumulation of multiple health deficits over the life course (Dent et al. 2019; Rockwood and Mitnitski 2007). Several studies have identified dyslipidemia as a risk factor for frailty and demonstrated the beneficial effects of lipid‐lowering drugs on frailty (Mak et al. 2023; Wang et al. 2019). Given the strong association between dyslipidemia and the development and severity of frailty, lifelong LDL‐C monitoring and lipid‐lowering strategies have been proposed as important approaches to reduce frailty and promote healthy aging (Mak et al. 2023).

Phytosterols are plant‐derived compounds structurally similar to cholesterol, primarily obtained from vegetable products. Due to their structural similarity to cholesterol, phytosterols competitively inhibit the intestinal absorption of dietary cholesterol. Daily consumption of 2–3 g of dietary phytosterols can reduce low‐density lipoprotein cholesterol (LDL‐C) levels by approximately 7%–10%, thereby promoting cardiovascular health (Mach et al. 2019). *The 2019 joint guidelines of the European Society of Cardiology and the European Society for Atherosclerosis* recommended that patients who have elevated LDL‐C levels but are not yet eligible for lipid‐lowering medications consume 2 g dietary phytosterols per day (Mach et al. 2019). However, the effect of phytosterols on frailty in blood, instead of intestine, remains uncertain. Mendelian randomization studies suggest that blood phytosterol levels are associated with an increased risk of major cardiovascular disease, mediated by non‐HDL cholesterol (Zhao et al. 2023; Scholz et al. 2022). Considering that cardiovascular disease is also a risk factor for frailty, we hypothesized that blood phytosterols may similarly influence frailty through non‐HDL‐C.

Given the potential association between blood lipids, blood phytosterol levels, and frailty, Mendelian randomization offers a powerful approach to elucidate the causal relationships among these factors. Mendelian randomization utilizes genetic variants that are randomly assigned at conception as proxies for exposures, thereby avoiding reverse causality and minimizing residual confounding. This approach provides a robust framework to disentangle the complex relationships between these biological factors and frailty, offering insights into potential causal pathways (Arsenault 2022; Holmes et al. 2017). Using Mendelian randomization, we aim to investigate the causal relationships between genetically proxied blood phytosterols and frailty. This methodology provides a platform to assess associations and examine the potential mediating role of lipid profiles in these relationships. By doing so, we aim to gain insights into the underlying mechanisms that may drive the observed associations, ultimately contributing to a more comprehensive understanding of frailty.

## 2 Method

### 2.1 Study Design and Database

G*uidelines for performing Mendelian randomization investigations (2023)* and *The STROBE‐MR Statement* were followed (Burgess et al. 2019; Skrivankova et al. 2021). The data sources for this study were derived from publicly available summary‐level data from genome‐wide association studies (GWAS), and detailed information about these datasets is summarized in Table S1. Since this study utilized publicly available, anonymized summary‐level data, separate ethical approval and individual consent were not required.

**FIGURE 1:**
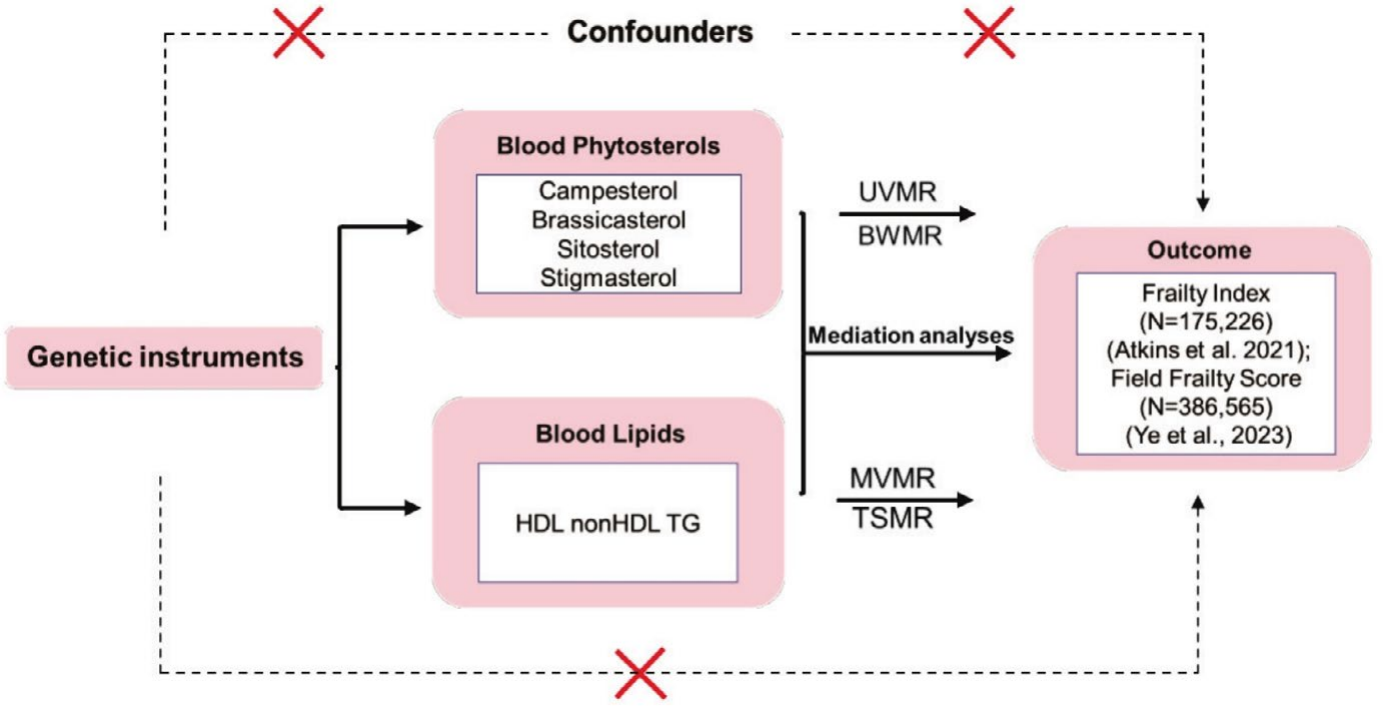
Outline of the study design. BWMR, Bayesian weighted Mendelian randomization; HDL‐C, high‐density lipoprotein cholesterol; MVMR, multivariate Mendelian randomization; TG, triglyceride; TSMR, two‐step Mendelian randomization; UVMR, univariate Mendelian randomization.

Figure 1 illustrates the study design. Genetic variants used in MR analyses satisfied the following assumptions: (1) association with the exposure, (2) independence from confounders, and (3) influence on the outcome solely through the exposure. To assess the causal influence of blood phytosterols on FI and FFS, we used univariate Mendelian randomization (UVMR). We also employed multivariate Mendelian randomization (MVMR) to evaluate the mediating role of blood lipids in the relationship between blood phytosterols and frailty.

For phytosterols, we used four phytosterol isoforms, including campesterol, brassicasterol, sitosterol, and stigmasterol (Scholz et al. 2022). These phytosterols were analyzed in a genome‐wide association study (GWAS) involving 9758 individuals from Iceland, utilizing liquid chromatography tandem mass spectrometry or gas chromatography mass spectrometry to quantify serum phytosterol levels.

Frailty Index data was leveraged from a GWAS meta‐analysis that combined data from the UK Biobank, comprising 164,610 participants of European ancestry aged 60–70 years, and the Swedish TwinGene study, with 10,616 participants aged 41–87 years (Atkins et al. 2021). Self‐reported questionnaires with similar content in the UK Biobank and TwinGene were collected and normalized by quartiles prior to analysis. The Fried Frailty Score (FFS) was collected from the UK Biobank of 386,565 participants of European ancestry and defined by five criteria (weight loss, exhaustion, physical activity, walking speed, and grip strength) (Ye et al. 2023).

As potential mediators of lipids, triglyceride (TG) and high‐density lipoprotein cholesterol (HDL‐C) summary‐level data were obtained from the 2013 Global Lipids Genetics Consortium (GLGC) to avoid weak instrument bias in MVMR with blood sitosterol. Summary levels of non‐HDL‐C and apolipoprotein B (APOB) were obtained from the GWAS of 115,078 Europeans from the UK Biobank.

### 2.2 Selection of Instrumental Variables

The selection of instrumental variables (IVs) must satisfy the three key assumptions of MR analysis: (1) IVs are associated with the exposure, (2) IVs are independent of confounders, and (3) IVs affect the outcome only through the exposure (Burgess et al. 2019; Skrivankova et al. 2021). Recommended independent genetic variations at genome‐wide level significance (*p* < 5 × 10^−8^) were identified as instrumental variables (IVs) for phytosterols (Zhao et al. 2023; Scholz et al. 2022). To address the potential weakness of IVs arising from a limited number of SNPs, we replicated the analyses for all blood phytosterols with a less strict standard of *p* < 5 × 10^−6^, LD: *r* ^2^ < 0.01 within 5 M bases. Genetic variations with *p* < 5 × 10^−8^ that are free of linkage disequilibrium (LD: *r* ^2^ < 0.001 within 10 M bases) were identified as IVs for lipids. No confounder related to outcomes was associated with IVs. *F*‐statistics of each IV were calculated as the square quotient of SNP‐exposure estimate and standard error and filtered with thresholds of 10 to avoid potential bias introduced by weak IVs (Bowden et al. 2016; Fu et al. 2024). Proxy single nucleotide polymorphisms (SNPs) with LD *r* ^2^ > 0.8 were used to replace missing IVs in the corresponding outcome. To minimize pleiotropy between blood sitosterol and mediators, mediator‐associated SNPs located on the same chromosome as sitosterol‐associated SNPs were excluded (Zhao et al. 2023).

### 2.3 Statistical Analysis

The inverse‐variance weighted (IVW) method was used to estimate the causal effect of genetically proxied blood phytosterols and lipids on frailty. Sensitivity analyses were conducted using MR‐Egger and weighted median methods. Heterogeneity and pleiotropy among SNPs were assessed using Cochran's *Q* test and the MR‐Egger intercept test (Burgess and Thompson 2017; Li et al. 2023; Verbanck et al. 2018). MR‐PRESSO was used to identify outliers in the results. Leave‐one‐out analysis was performed to evaluate the robustness of the causal effects by sequentially excluding each SNP. Bayesian Weighted Mendelian Randomization (BWMR) was used for offering enhanced resistance to weak instrument bias and horizontal pleiotropy (Zhao et al. 2020). Given the similarity in describing frailty and the differences in concepts, we chose FI as primary analyses and FFS as replication analyses. False discovery rate (FDR) method was applied for multiple testing in primary and replication analyses.

Multivariate Mendelian randomization (MVMR) analysis was used to assess the mediating role of lipids in the relationship between phytosterols and FI, with both phytosterols and lipids included as exposures. The two‐step Mendelian randomization (TSMR) approach was employed to separately estimate: (1) the exposure → mediator effect and (2) the mediator→outcome effect. TSMR can explicitly decompose the total effect of phytosterols into an indirect effect, which follows the pathway of phytosterols → lipids → frailty, and a direct effect, which is the pathway of phytosterols → frailty, independent of lipids. Indirect effects and mediation proportions were estimated using the product of coefficients method. Standard errors for indirect effects were obtained using the delta method.

To validate the directionality of the observed causal relationships, we performed bidirectional Mendelian randomization analysis. IVs for FI were selected based on genome‐wide significant variants (*p* < 5 × 10^−8^) that were independent of linkage disequilibrium (LD) (*r* ^2^ < 0.001 within a 10 Mb window). The outcomes of the bidirectional analysis included blood sitosterol, LDL, non‐HDL‐C, and ApoB.

All statistical analyses were conducted using the “TwoSampleMR,” “MendelianRandomization,” “MVMR,” “BWMR,” and “MR‐PRESSO” packages in R (version 4.3.2).

## 3 Results

The number of instrumental variables (IVs) identified for blood brassicasterol, campesterol, sitosterol, and stigmasterol was 3, 4, 7, and 3, respectively (Table S2). The *F*‐statistic values for all IVs exceeded the threshold of 10, indicating a low risk of weak instrument bias (Tables S2 and S7).

**FIGURE 2:**
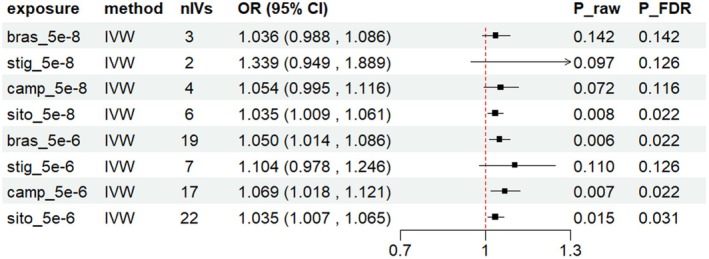
Associations between genetic proxied blood phytosterols and Frailty Index. Genetic proxied blood sitosterol was associated with an increased risk of higher Frailty Index at all IVs; standard, campesterol, and brassicasterol were only positive at 5e‐6. nIVs, number of instrumental variables; OR, odds ratio.

**FIGURE 3:**
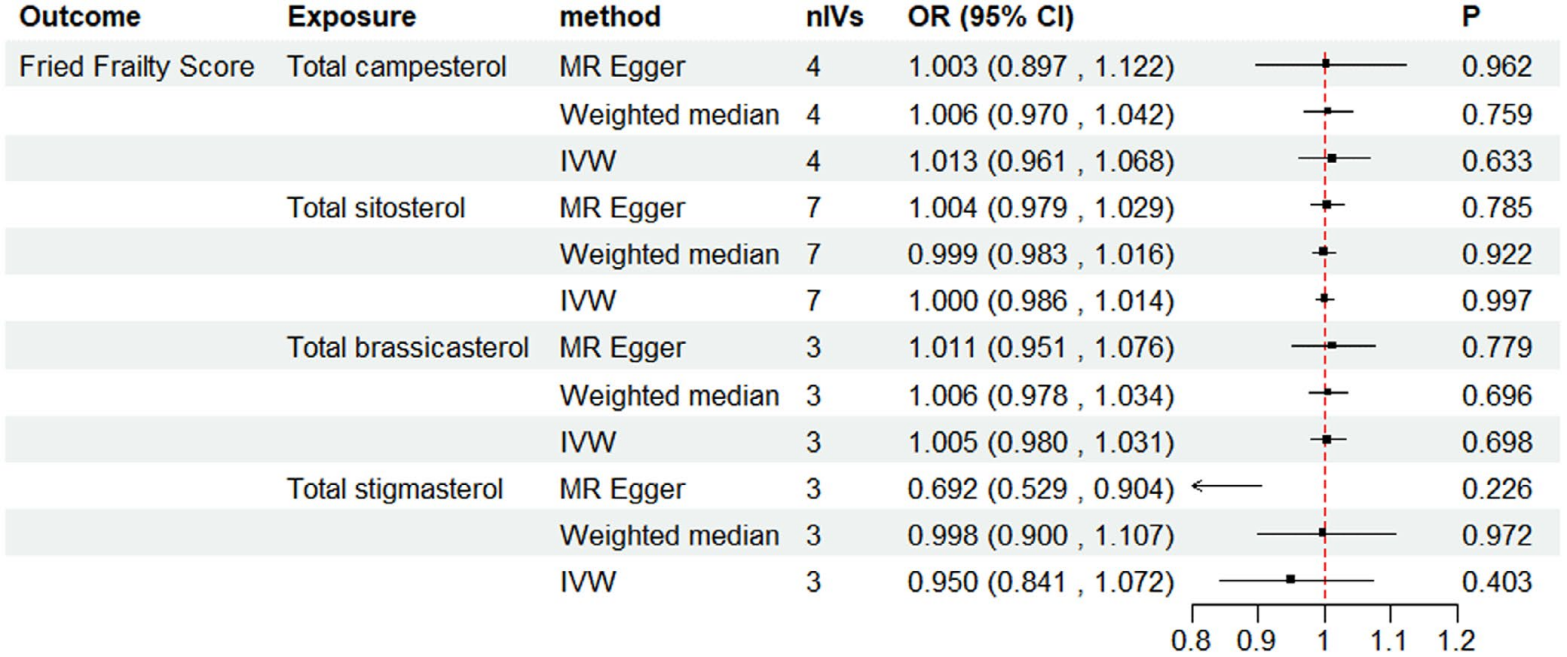
Associations between genetic proxied blood phytosterols and Fried Frailty Score. No association was found between genetic proxied blood sitosterol and FFS. nIVs, number of instrumental variables; OR, odds ratio.

Using the IVW method, blood sitosterol was associated with a higher Frailty Index [OR, 95% CI = 1.035 (1.009, 1.061), *p* = 0.008] (Figure 2). Sensitivity analyses using MR‐Egger and weighted median methods yielded consistent results. MR‐Egger regression showed no evidence of horizontal pleiotropy (Table S4). No outliers were detected by MR‐PRESSO (Table S10). Leave‐one‐out analysis demonstrated the robustness of the UVMR results, showing consistent effects of the remaining SNPs on the outcome after sequentially excluding each SNP (Table S5). In replication analyses, total brassicasterol [OR, 95% CI = 1.050 (1.014, 1.086), *p* = 0.006], total campesterol [OR, 95% CI = 1.069 (1.018, 1.121), *p* = 0.007], total sitosterol [OR, 95% CI = 1.035 (1.007, 1.065), *p* = 0.015] showed a causal relationship with FI separately. The BWMR results were consistent with the analysis (Table S13), further confirming the robustness of our research findings. Among these phytosterols, sitosterol showed a causal effect on a higher FI in all IVs standard, while in lower IVs standard, brassicasterol and campesterol also showed significant effects in the same direction. This suggests that the promoting effect of genetic prediction on FI may not be specific to sitosterol, but rather a shared effect of blood phytosterols. No significant association was observed between blood phytosterols and the Fried Frailty Score (Figure 3, Table S4). This suggests that the effect of blood phytosterols may underlie the differences between FI and FFS, particularly through their association with blood lipids.

**FIGURE 4:**
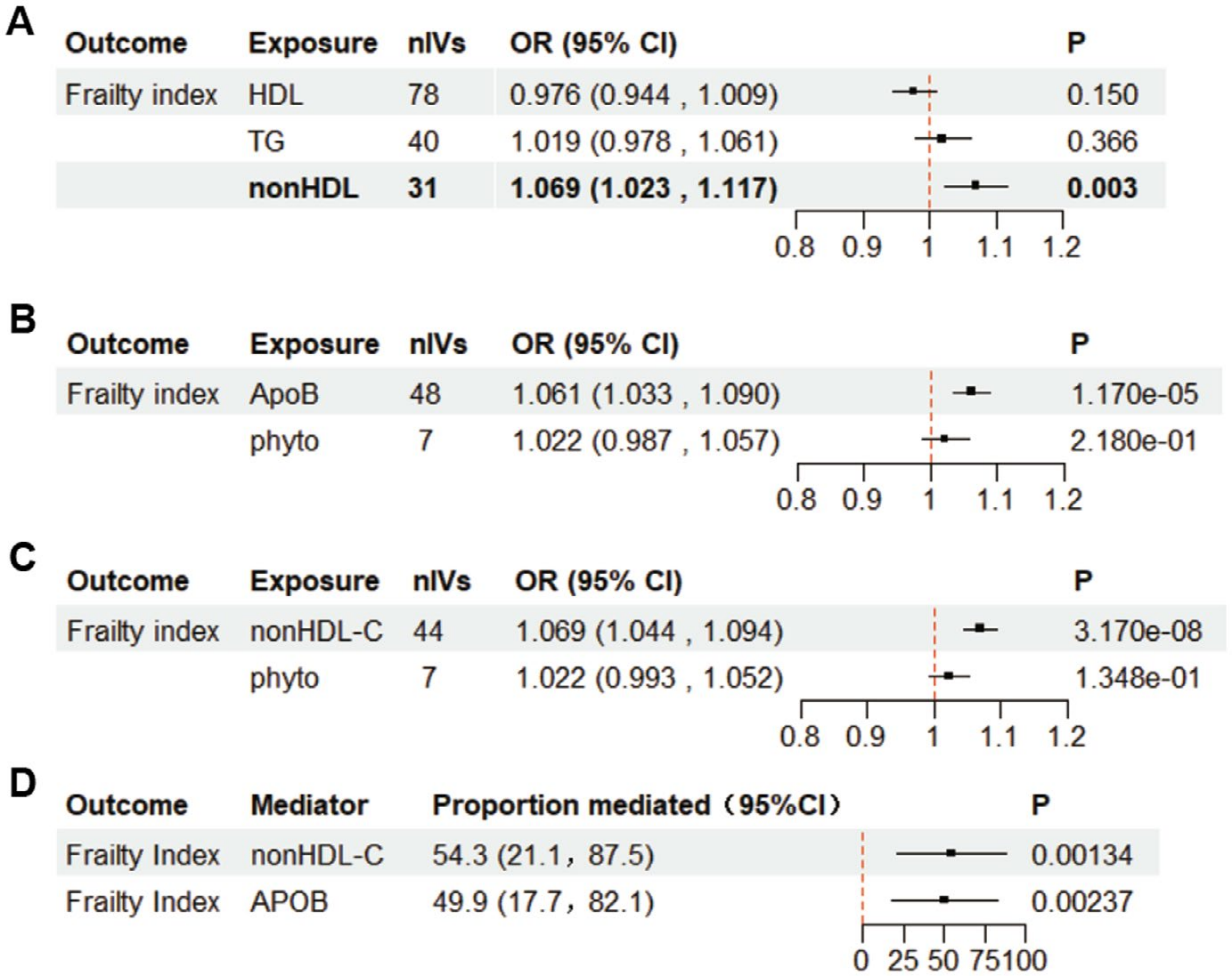
(A) Multivariable‐adjusted associations between blood lipid and Frailty Index. Effects of blood lipid levels on the risk of Frailty Index in multivariable Mendelian randomization. Non‐HDL‐c mediated the direct effects on the Frailty Index. (B, C) Multivariable‐adjusted associations between blood sitosterol and Frailty Index. After multivariate adjustment for APOB, non‐HDL‐c as an exposure factor, the causal estimate of blood sitosterol on the Frailty Index disappeared. (D) Non‐HDL‐C and APOB mediating proportions in the effect of blood sitosterol on frailty. HDL‐C high‐density lipoprotein cholesterol; MVMR, multivariable Mendelian randomization; OR, odds ratio; TG, triglycerides.

To further analyze the reasons for the difference between FI and FFS results, we conducted multivariate Mendelian randomization (MVMR) analyses to evaluate the potential mediating role of blood lipids in the relationship between sitosterol and FI. First, we assessed the causal relationship between blood lipids (HDL‐C, TG, and non‐HDL‐C) and the Frailty Index. The results indicated that blood non‐HDL‐C primarily drove the causal effect of blood lipids on the Frailty Index [OR, 95% CI = 1.069 (1.023, 1.117), *p* = 0.003] (Figure 4).

Based on previous studies, blood non‐HDL‐C and APOB were included as exposure factors, as APOB is a carrier of non‐HDL‐C and LDL‐C in the blood (Zhao et al. 2023). MVMR analyses revealed that the effect of blood sitosterol on the Frailty Index was attenuated after adjusting for blood non‐HDL‐C and apolipoprotein B (Figure 4B,C). Blood non‐HDL‐C and APOB mediated [54.3% (21.1%, 87.5%), *p* = 0.00134] and [49.9% (17.7%, 82.1%), *p* = 0.00237] of the effect of phytosterols on the Frailty Index, respectively (Figure 4D). The two‐step MR mediation analysis yielded consistent results, with mediation proportions of [58.06% (95% CI: 21.71%, 94.42%), *p* = 0.0017] and [33.92% (95% CI: 7.15%, 60.68%), *p* = 0.013], respectively (Table S12). The bidirectional Mendelian randomization analysis revealed no evidence of reverse causation after excluding pleiotropic SNPs (Table S11). These findings suggested that the effects of sitosterol on the Frailty Index may be mediated by blood lipids, particularly non‐HDL‐C. Notably, due to the high biological correlation between non‐HDL‐C, APOB, and LDL in lipoprotein metabolism and pathological mechanisms, their individually estimated mediation effects may include shared variance, potentially leading to a cumulative proportion exceeding 100%.

## 4 Discussion

In this study, we examined the association between blood phytosterols and both the Frailty Index (FI) and Fried Frailty Score (FFS). Although the effect size is only about 1/6–1/3 of that of LDL‐C's impact on the FI (Wang et al. 2019), we identified a mild causal association between blood phytosterols and the Frailty Index, but no significant association with the FFS. Furthermore, blood non‐HDL‐C and APOB may mediate the relationship between blood sitosterol and the FI. This will facilitate our investigation into the shared pathophysiological mechanisms through which these cholesterol‐related substances contribute to the development of frailty.

Hyperphytosterolemia, a rare genetic disorder caused by inactivating mutations in the ABCG5/8 genes, is associated with an increased risk of cardiovascular disease (Stonehouse et al. 2025). It has been hypothesized that phytosterols may exert effects similar to cholesterol in the vascular system (Zhao et al. 2023). A Mendelian randomization study indicated that even within the normal range, elevated blood phytosterol levels increase the risk of cardiovascular disease (a 6% higher risk per 20% increase in blood sitosterol), with approximately half of this effect mediated by non‐HDL‐C (Zhao et al. 2023). Both cardiovascular disease and elevated cholesterol levels are risk factors for frailty, and long‐term lipid control is recognized as beneficial for reducing frailty (Wang et al. 2019). The effects of dietary phytosterol supplementation on lipids and cardiovascular events remain controversial. Although intestinal absorption of phytosterols has been linked to increased atherosclerosis and cardiovascular risk, even within the normal range (Zhao et al. 2023; Rocha et al. 2016), the clinical benefits of phytosterol supplementation are debated. Conversely, the efficacy of phytosterol supplements in lowering LDL‐C has been questioned in clinical studies (Ruscica et al. 2025). A combination drug containing 1.8 g/day of phytosterols showed disappointing lipid‐lowering effects in a recent 4‐month randomized double‐blind placebo‐controlled trial (Stonehouse et al. 2025), but phytosterols showed effectiveness in a meta‐analysis of several RCTs (Rocha et al. 2016). Phytosterols inhibit cholesterol absorption by competing for intestinal absorption via the same pathway in the intestine because of their similar chemical structure and may be involved in the transcriptional induction of genes involved in cholesterol metabolism (Ruscica et al. 2025). However, its lipid‐lowering effect depends on the body's cholesterol synthesis pathway, and when cholesterol is primarily synthesized by the liver rather than absorbed intestinally, the effect of dietary phytosterols may be disappointing (Ruscica et al. 2025). When intestinal cholesterol absorption is increased by genetic variants in NPC1L1 and ABCG5/8, these dietary phytosterols lead to increased blood phytosterol levels and similarly increased risk of disease, thus making the risk of dietary phytosterol use a question awaiting evaluation (Stellaard and Lütjohann 2025). The modest effect size of blood sitosterol on FI and the neutral effect on FFS in our study suggest that further evidence is needed to establish blood phytosterols as a risk factor for frailty.

The Frailty Index evaluates frailty based on 49 health deficits across 11 domains, including sensory, cranial, mental well‐being, infirmity, cardiometabolic, respiratory, musculoskeletal, immunological, cancer, pain, and gastrointestinal health (Atkins et al. 2021). The Fried Frailty Score assesses frailty across five dimensions: fatigue, weight loss, walking speed, grip strength, and physical activity (Ye et al. 2023). On one hand, when considering the differences in Mendelian Randomization (MR) results between the Frailty Index (FI) and the Frailty Phenotype Score (FFS), the influence of residual confounding factors cannot be overlooked, such as measurement errors in physical activity levels or components derived from self‐reported data. On the other hand, these differences may reflect the distinct domains captured by these two different frailty measures. Cardiovascular diseases (CVDs) serve as a critical outcome influenced by lipid‐related traits, act as a bidirectional risk factor for frailty (James et al. 2024), and represent the primary therapeutic target of phytosterol‐based lipid‐lowering strategies. Notably, CVDs also underlie key distinctions between the FI and the FFS (cardiometabolic factors). These lines of evidence collectively suggest that cardiometabolic factors may mediate a portion of the association between blood phytosterols and FI, thereby providing a plausible explanation for the observed discrepancies in how phytosterols relate to FI versus FFS.

Our findings are consistent with previous studies, demonstrating a positive causal relationship between blood phytosterol and non‐HDL‐C levels (Zhao et al. 2023). Due to the shared absorption pathways of phytosterols and cholesterol, hypercholesterolemia is often associated with elevated blood phytosterol levels, even in patients without hyperphytosterolemia (Zhao et al. 2023; Huang et al. 2022). The overlapping effects of phytosterols on frailty and cardiovascular disease may suggest shared mechanisms, particularly those mediated by lipids. Indeed, the bidirectional relationship between frailty and cardiovascular disease has long been widely observed (James et al. 2024). Modified lipoproteins, such as oxidized LDL (oxLDL), may trigger chronic inflammatory and immune responses, contributing to both cardiovascular disease and frailty through shared inflammatory markers, including interleukin‐6 (IL‐6) and tumor necrosis factor‐alpha (TNF‐α) (James et al. 2024; Engelen et al. 2022; Bielecka‐Dabrowa et al. 2020). However, the effects of phytosterols on these inflammatory markers remain inconsistent across studies. In particular, all the beneficial effects seen at the experimental level are strongly inconsistent with clinical observations and applications (Vilahur et al. 2019). For example, some studies predicted a potential target of phytosterols, nuclear factor erythroid 2‐related factor 2 (Nrf2), and attenuated NLR family pyrin domain containing 3 (NLRP3)‐associated inflammation and disease through this pathway in animal models (Soni et al. 2025; Ding et al. 2025). However, in human clinical applications and studies, in a systematic review and meta‐analysis including 20 RCTs involving 1308 subjects, it was shown that phytosterol intake affects absolute changes in plasma C‐reactive protein (CRP), but the effect on low‐grade inflammation remains unclear (Rocha et al. 2016). These experimental findings require additional research to reinforce the clinical benefits, including harmonizing the metrics used to study inflammation.

Blood phytosterol levels have traditionally been used as markers of cholesterol absorption (Scholz et al. 2022). In this study, blood phytosterol concentrations were identified as a potential risk factor for frailty, adding to their established role as cholesterol absorption markers. Particularly, patients with hereditary hypercholesterolemia should consider the potential health risks associated with elevated blood phytosterol levels when opting for phytosterol‐based lipid‐lowering strategies.

## 5 Limitations

First, this study focused on the effects of blood phytosterols on frailty and did not assess the overall impact of dietary phytosterol intake on frailty. Although blood phytosterols may modestly increase the risk of Frailty Index, dietary phytosterol intake could potentially reduce risk by lowering non‐HDL‐C levels. Further clinical trials are needed to explore these issues. Sensitivity analyses showed consistent trends across different MR methods, indicating robust results. Neither the MR‐Egger intercept nor the MR‐PRESSO test detected significant pleiotropy or heterogeneity, supporting the validity of our causal inferences. Although we employed multiple methods to test the MR assumptions, we cannot completely rule out potential violations, particularly undetected pleiotropy and unmeasured confusion between mediator and outcome, which may confound our results. Our study was limited to participants of European ancestry, highlighting the need for future research to assess the generalizability of our findings to other populations. Additionally, the use of summary‐level data limits our ability to explore potential nonlinear relationships or subtype‐specific effects between phytosterols and frailty. The GWAS summary‐level data of non‐HDL‐C cholesterol and ApoB were derived from UK Biobank, which may partially overlap with the data sources used for FI and FFS analyses. This potential sample overlap could introduce bias into our estimates. To ensure robust instrument strength and avoid potential issues with conditional F‐statistics for sitosterol, we intentionally selected lipid trait data from the 2013 GLGC consortium rather than the 2021 version. While the 2013 dataset contains approximately 4% participants of non‐European ancestry, we recognize that ancestral differences in allele frequencies and linkage disequilibrium patterns could potentially influence MR validity. We emphasize that future studies utilizing more diverse genetic datasets would be valuable to verify the generalizability of our findings. Although FFS did not show significant results, heterogeneity between the FFS and FI populations may confuse the results.

## 6 Conclusion

This study provides evidence for a modest association between blood phytosterols and an increased risk of FI. Furthermore, blood non‐HDL‐C and ApoB may mediate a significant proportion of the association between blood sitosterol and FI. The differential effects of blood phytosterols on FI and FFS outcomes may indicate a mediating role of specific health deficits, particularly cardiometabolic factors.

## Author Contributions

**Guangyu Gao:** conceptualization (equal), writing – original draft (equal). **Tianci Yao:** methodology (lead), writing – review and editing (equal). **Chengyun Liu:** supervision (lead), writing – review and editing (equal). **Haohui Fan:** formal analysis (equal). **Xinyue Zhang:** data curation (equal). **Hao Zhang:** software (equal). **Xiaofang Zhao:** investigation (equal). **Bei Song:** investigation (equal). **Kun Wang:** resources (equal). **Ting Liu:** data curation (equal), visualization (equal). **Xueke Guang:** data curation (equal), visualization (equal). **Quan Zhou:** data curation (equal), visualization (equal). **Weilin Lu:** conceptualization (equal), supervision.

## Conflicts of Interest

The authors declare no conflicts of interest.

## Supporting information


Table S1.

## Data Availability

Full GWAS summary statistics are freely available to download via the GWAS catalog (https://www.ebi.ac.uk/gwas/downloads/summary‐statistics) or ieu OpenGWAS project (https://gwas.mrcieu.ac.uk/). glgc‐lipids2021 GWAS summary statistics are from https://csg.sph.umich.edu/willer/public/glgc‐lipids2021/. Blood phytosterol GWAS summary statistics are from https://doi.org/10.1038/s41467‐021‐27706‐6.
